# Timeline of Changes in Biomarkers Associated with Spinal Cord Injury–Induced Polyuria

**DOI:** 10.1089/neur.2021.0046

**Published:** 2021-10-27

**Authors:** Jason H. Gumbel, Cui Bo Yang, Charles H. Hubscher

**Affiliations:** ^1^ Department of Anatomical Sciences and Neurobiology, University of Louisville, Louisville, Kentucky, USA.; ^2^ Kentucky Spinal Cord Injury Research Center, University of Louisville, Louisville, Kentucky, USA.

**Keywords:** hypothalamus, kidney, natriuretic peptides, polyuria, suprachiasmatic nucleus, vasopressin

## Abstract

Deficits in upper and lower urinary tract function, which include detrusor overactivity, urinary incontinence, detrusor-sphincter dyssynergia, and polyuria, are among the leading issues that arise after spinal cord injury (SCI) affecting quality of life. Given that overproduction of urine (polyuria) has been shown to be associated with an imbalance in key regulators of body fluid homeostasis, the current study examined the timing of changes in levels of various relevant hormones, peptides, receptors, and channels post-contusion injury in adult male Wistar rats. The results show significant up- or downregulation at various time points, beginning at 7 days post-injury, in levels of urinary atrial natriuretic peptide, serum arginine vasopressin (AVP), kidney natriuretic peptide receptor-A, kidney vasopressin-2 receptor, kidney aquaporin-2 channels, and kidney epithelial sodium channels (β- and γ-, but not α-, subunits). The number of AVP-labeled neurons in the hypothalamus (supraoptic and -chiasmatic, but not paraventricular, nuclei) was also significantly altered at one or more time points. These data show significant fluctuations in key biomarkers involved in body fluid homeostasis during the post-SCI secondary injury phase, suggesting that therapeutic interventions (e.g., desmopressin, a synthetic analogue of AVP) should be considered early post-SCI.

## Introduction

Spinal cord injury (SCI) results in deficits that are widespread across all bodily systems, including those involving motor, sensory, autonomic, endocrine, and immune functions. Urological dysfunctions are rated by SCI persons as one of the most important factors impacting quality of life.^1^ However, the vast majority of pre-clinical and clinical studies targeting the urinary system have focused on the lower urinary tract (bladder, urethra, and external urethral sphincter), with relatively fewer studies addressing deficits related to the upper urinary tract (kidney) post-SCI. A common issue that arises after SCI that complicates urinary function is the excess production/passage of urine (polyuria/nocturia),^2^ which has been reported clinically and in pre-clinical animal models.^3–6^ The frequency of daily bladder catheterizations, including during the night-time, is disruptive to daily activities and sleep while simultaneously increasing the risk of urinary tract and bladder infections.^7^ However, many persons with SCI accommodate by limiting their intake and/or type of fluids, which complicates the estimation of polyuria prevalence and can lead to dehydration or other issues impacting health.^8^

Our group has previously shown, using a pre-clinical animal incomplete contusion model, that 1) polyuria occurs regardless of severity of SCI,^6^ 2) alterations of hormones affecting diuresis are present at 14 days post-injury (dpi),^9^ and 3) related receptor/channel densities are altered in the kidney at 10 weeks post-SCI.^10^ Although several key hormones and their receptors in the kidney impacting fluid balance have been shown to contribute to the mechanisms underlying SCI-induced polyuria/nocturia, more information is needed for the optimization of therapeutic approaches.^9,10^

Several studies have already been published to date demonstrating significant disruptions in arginine vasopressin (AVP; also commonly referred to as antidiuretic hormone), atrial natriuretic peptide (ANP), and their associated receptors (vasopressin-2 receptor [V2R] and natriuretic peptide receptor-A [NPRA], respectively) in the kidney after SCI.^9–11^ We have also shown that kidney aquaporin-2 (AQP2) channels and epithelial sodium channels (ENaCs) were significantly decreased post-SCI in adult male rats.^10^ Together, these fluctuations in AVP and ANP and their accompanying receptors/channels lead to an increase in urine production based on their physiological function. These receptors also play major roles in salt and fluid balance within the body, as well as cardiovascular homeostasis.^12,13^ Clinically, desmopressin (synthetic AVP) may be used to treat nocturnal polyuria, but with limited results and potential deleterious side effects.^14,15^ Additionally, desmopressin often impacts blood pressure, which may increase bouts of autonomic dysreflexia—a phenomenon that occurs when blood pressure rises uncontrollably and cannot be corrected because of disruptions of descending sympathetic pathways in the spinal cord.^16,17^

The purpose of the current study was to ascertain a timeline for the development and maintenance of SCI-induced polyuria, with a focus on contributions of key targets not only in the kidney, but also within the hypothalamus. Neurons in the hypothalamus manufacture AVP in the supraoptic nucleus (SON), paraventricular nucleus (PVN), and the suprachiasmatic nucleus (SCN).^18^ Under normal physiological circumstances, AVP is released by the posterior pituitary in response to hypotension and/or an increase in extracellular osmolarity. In the kidney, AVP activates V2R to increase AQP2 channels for the reabsorption of solute-free water back into the bloodstream. An increase in blood volume will trigger the release of ANP from the heart and, within the kidney, will activate NPRA in order to increase the excretion of salt and inhibit the reabsorption of water.^19,20^ Understanding the timeline of when these key biomarkers are significantly altered post-injury will provide a therapeutic window for intervention.

## Methods

### Animals

All animal experiments and procedure protocols were reviewed and approved by the University of Louisville Institutional Animal Use and Care Committee and carried out in accordance to the National Institutes of Health (NIH) guidelines. A total of 94 adult male Wistar rats were used for the entirety of these experiments (Table 1). We have opted to utilize only adult male rats for the current study given that prevalence of SCI is predominantly male. Six timeline SCI groups included study end-points at 3, 7, 14, 28, 42, and 84 days post-injury (dpi) as well as three surgical sham groups matched for end-points 3, 14, and 42 dpi.

**Table 1.** SCI Impactor Parameters and Assessment Outcome Values

Group	n	Injury force (kdyne)	Displacement (μm)	4-day urine	7-dpi BBB	Terminal BBB
Sham	28^a^	—	—	0	20.10 ± 0.74	19.80 ± 0.83
3 dpi	12	223.4 ± 15.2	1367.4 ± 124.5	—	—	4.30 ± 2.63^b^
7 dpi	10	244.4 ± 34.5	1330.8 ± 311.7	3.95 ± 0.66	6.00 ± 0.78	6.00 ± 0.78^b^
14 dpi	11	222.7 ± 3.8	1378.5 ± 134.1	3.31 ± 1.07	7.60 ± 0.20	8.40 ± 1.57
28 dpi	11	224.3 ± 13.7	1427.8 ± 139.1	3.50 ± 1.07	7.40 ± 2.25	9.60 ± 1.57
42 dpi	10	238.5 ± 33.3	1501.7 ± 79.8	1.86 ± 1.16	5.20 ± 2.93	9.40 ± 1.35
84 dpi	12	237.3 ± 35.3	1494.4 ± 162.3	4.25 ± 1.11	6.40 ± 2.43	9.90 ± 1.24

Values indicated are mean ± standard deviation; all SCI groups are significantly different (*p* < 0.01) from sham (4-day urine; 7-dpi and terminal BBB).

^a^ Data collected from three sham animal groups were combined because no significant differences were found.

^b^ Both the 3- and 7-dpi injured groups received significantly lower scores than 14-, 28-, 42-, and 84-dpi groups (*p* < 0.05).

SCI, spinal cord injury; dpi, days post-injury; BBB, Basso-Beattie-Bresnahan.

### Spinal cord injury

After baseline/pre-injury data were obtained (see below), animals were anesthetized with an intraperitoneal injection of a mixture of ketamine (80 mg/kg, Ketoset^®^; Fort Dodge Laboratories, Fort Dodge, IA) and xylazine (10 mg/kg, AnaSed; Lloyd Laboratories, Shenandoah, IA). Both toe-pinch and orbital reflexes were monitored to assure that a deep anesthetic plane was reached. The surgical site was shaved and cleansed with chlorhexidine scrub 4% (Henry Schein) before placement on a heating pad and application of sterile ocular lubricant (OptixCare; Aventix, Burlington, Ontario, Canada). A T8 laminectomy was performed to expose the T9 spinal cord level. An Infinite Horizon (IH) impactor (Precision Systems and Instrumentation, LLC; Fairfax Station, VA) was used to administer a 215-kilodyne contusion with no dwell time at the T9 spinal level, which yields a moderate-severe extent of injury with white matter sparing (WMS) averaging ∼11%.^6,21^ The muscular layer was then sutured with a 4-0 surgical suture (Ethicon Inc., Somerville, NJ) and the skin closed with surgical wound clips. Surgical sham animals underwent the same procedure, but no contusion injury was administered.

Both gentamicin sulfate (GentaFuse; Henry Schein Animal Health, Dublin, OH) and meloxicam (Eloxiject; Henry Schein Animal Health) were injected subcutaneously per established post-operative care procedures.^10,22,23^ Physiological saline was also administered post-operatively (5 mL before contusion and 5 mL after contusion). Bladder emptying by the Crede maneuver was performed three times daily until individual animals reached reflexive bladder function (by 6 days^24,25^). Animals were closely checked at these times for any signs of distress or dehydration. Residual volumes at 4 dpi were collected and measured for use as an indicator of lesion severity, per our previous study showing peak amounts at this time point attributable to flaccid paralysis from spinal shock during the earliest phase post-injury.^6^ Note that the injury procedure is available for online viewing in a published video journal from our lab.^23^

### Metabolic cage data collection

A six-station Comprehensive Lab Animal Monitoring System (CLAMS; Columbus Instruments, Columbus, OH) was used to monitor 24-h urine output volume and drink volume, following established protocols.^6,10,26^ Food and water were available *ad libitum*. As part of the acclimation procedure of animals to the CLAMS unit, pre-injury baseline data were collected twice in 1 week, but only the second 24-h period was used for analysis. Metabolic cage assessments were then carried out once-weekly after SCI (number of times variable between time-course groups).^9,10,23^

### Blood sample collections

For serum samples, blood was collected by the lateral tail vein at pre-injury and terminal time points, following standard procedures.^9,27^ Animals were anesthetized with isoflurane before shaving the base of the tail to better visualize the lateral tail veins. Once the animals were placed on a heating pad, an 18-g needle was used to puncture either of the lateral tail veins. Between 0.5 and 0.7 mL of venous blood was then collected into serum separator tubes (BD microcontainer; Becton, Dickinson and Co., Franklin Lakes, NJ). Light pressure was applied to the puncture site with 2” × 2” gauze until bleeding stopped. When necessary, wound clotting was achieved using styptic powder with benzocaine (Kwik-Stop; ARC Laboratories). Blood samples were centrifuged at 14,000 rpm for 15 min, and serum was collected and frozen at −20°C.

### Locomotor assessment

The Basso-Beattie-Bresnahan (BBB) open field locomotor test^28^ was performed weekly on each rat as well as the day before termination and subsequent tissue removal. Each single score per animal represents an average of the left and right hindlimb BBB score assigned by two experimenters blinded to time-point status. This assessment is used as an additional indicator of lesion severity and spontaneous recovery, per our previously published data.^6^

### Tissue collection and histology

On the specified terminal date (3, 7, 14, 28, 42, and 84 dpi/sham laminectomy), animals were perfused transcardially with a solution of heparinized saline (1 mL of heparin/100 mL of normal saline), followed by 4% paraformaldehyde (PFA). Although multiple tissues were retrieved, only the brain, spinal cord (lesion site), and left kidney were removed and utilized in this study for immunohistochemistry (IHC), contusion epicenter reconstruction, and western blot analysis, respectively. Note that the kidney was retrieved before the 4% PFA perfusion for western blot assessments. Each kidney was cut to ∼0.1-g sections (including both medulla and cortex) before storage at −80°C.

The brain and spinal cord were submerged in 4% PFA and stored for at least 24 h at 4°C. Tissue was then moved to a 30% sucrose solution and stored at 4°C until tissue had sunk to the bottom of the 15-mL conical tube (24–48 h) and stored until it was sectioned using a cryostat (Leica CM 1850; Leica Biosystems, Wetzlar, Germany). Tissue was frozen in tissue-freezing medium at −80°C to prevent freezing artifact. Once ready to section, tissue was acclimated to −20°C inside the cryostat for at least 30 min. Hypothalamus sections were collected at serial 25-μm sections and transferred to glass slides. Slides were then stored at 4°C until tissue was ready to be stained.

IHC was performed on tissue sections containing the hypothalamus to visualize and quantify AVP-positive neurons. Fixed tissue was washed in phosphate-buffered saline before unmasking epitopes using antigen retrieval (IHC antigen retrieval reagent citrate, pH 6.0; Enzo Life Sciences, Farmingdale, NY). After antigen retrieval, tissue sections were washed and blocked (SuperBlock Blocking Buffer; ThermoFisherScientific, Waltham, MA) before applying primary antibodies. Tissue sections were incubated with primary antibody antivasopressin (1:1000 dilution; ab39363; Abcam, Cambridge, MA) overnight at 4°C. Slides were then washed before incubation with fluorescent-conjugated goat antirabbit secondary antibody (Alexa Fluor 488; ThermoFisher Scientific). Slides were again washed before counterstaining with 4′,6-diamidino-2-phenylindole to visualize nuclei.

To quantify the number of AVP-labeled neurons in the hypothalamus, the SON, SCN, and PVN were targeted based upon regional landmarks identified in the Rat Brain Atlas.^29^ A block of tissue from the collected brain was formed rostral to the optic chiasm and rostral to the cerebellum. Once frozen, tissue sections were cut at 25-μm thickness and each slide that was stained contained three tissue sections ≥75 μm apart to ensure that cells were not counted more than once. Slides were coded by an experimenter not directly involved in the study to blind group identity. Using ImageJ (NIH, Bethesda, MD), the area of the SON, SCN, or PVN was outlined, and the number of AVP-positive cells with an intensity threshold ≥1.5 times above background level were counted to obtain the cells per area for quantification. At least four different sections of nuclei per animal were averaged together for analyses.

Spinal lesion histology was carried out as previously described.^10,26,30^ The spinal cord area containing the lesion (including approximately two levels above and below) was sectioned at 20-μm thickness and stained with Luxol fast blue and cresyl violet. The lesion epicenter and WMS was captured and analyzed using Spot Advanced software (Diagnostic Instruments, Sterline Heights, MI) and Nikon E400 microscope (Nikon Corporation, Tokyo, Japan). Percent WMS was calculated by dividing the intact white matter at the lesion epicenter by the average area of intact white matter present in more intact sections both rostral and caudal the injury site. An average of two areas within 2 mm rostral and caudal was used for intact white matter.^31^

### Enzyme-linked immunosorbent assay

Urinary ANP and creatinine was measured as previously described^10^ using an ANP Enzyme Immunoassay Kit (catalog no. K026-H1; Arbor Assays, Ann Arbor, MI). Urine samples were diluted 1:5 for ANP and 1:20 for creatinine, then plated in a 96-well plate in duplicate. Plates were read at a 450-nm optical density using SoftMax Pro software (Molecular Devices, LLC, San Jose, CA). Urinary creatinine was measured using the DetectX Urinary Creatinine Detection Kit (catalog no. K002-H5; Arbor Assays). To control for differing urinary concentrations, urinary ANP levels were divided by urinary creatinine levels per ANP enzyme-linked immunosorbent assay (ELISA) kit instructions.

Baseline and terminal levels of serum AVP were determined using an arginine vasopressin ELISA kit (cat. no. OKEH02585; Aviva Systems Biology Corporation, San Diego, CA). Stored serum samples (see above) were diluted at 1:5, and ELISA was carried out according to kit instructions.

### Western blot

Relative expression of AVP, NPRA, AQP2, and ENaC (α-, β-, and γ-subunits) was analyzed by western blot analysis according to our previously published protocols.^9,10^ Kidney tissue was homogenized in ice-cold radioimmunoprecipitation assay buffer (R0278; Sigma-Aldrich, St. Louis, MO) and protease inhibitor (78425; ThermoFisherScientific). Concentration of protein was determined for each sample using Bradford protein assay reagent (5000201; Bio-Rad Laboratories, Hercules, CA) and a spectrophotometer (at 595-nm absorbance). Gels were then run by loading 50 μg of protein per lane on a 4–15% gradient gel (456-1085, miniprotean TGX gels; Bio-Rad) at 100 V for 80 min in miniprotean gel tanks (running buffer was a 1 × Tris/glycine/sodium dodecyl sulfate buffer). Protein was transferred to polyvinylidene difluoride membranes for 2 h in 4°C at 80 V and stained with Ponceau S to visualize bands. Membranes were washed with 1 × Tris-buffered saline with Tween 20 (TBST), then blocked in 5% non-fat dry milk TBST solution for 1 h. Membranes were then incubated in the primary antibody at 4°C overnight.

Antibodies used in this study included anti-NPRA (dilution 1:1000, ab154280; Abcam), anti-AVP receptor V2 (dilution 1:750, ab108145; Abcam), anti-AQP2 (dilution 1:1000, ab108065; Abcam), anti-ENaC α-subunit (dilution 1:1000, SPC-403; StressMarq Biosciences, Victoria, British Columbia, Canada), anti-ENaC γ-subunit (dilution 1:1000, SPC-405; StressMarq Biosciences), anti-ENaC β subunit (dilution 1:750, 14134-1-P; Proteintech, Sankt Leon-Rot, Germany), and anti-β-actin (dilution 1:5000, #A5316; Sigma-Aldrich). Membranes were washed three times with TBST before incubation with horseradish peroxidase–conjugated secondary antibody in blocking solution for 2 h at 1:3000. Last, membranes were washed three times with TBST and developed with enhanced chemiluminescence substrate before being imaged using the Bio-Rad Imaging System and analyzed using ImageJ software (version 1.8; NIH). Standardized values were obtained by normalizing experimental groups to β-actin.

### Statistical analysis

Both 24-h urine and drink volumes from CLAMS data were exported from Oxymax software to Microsoft Excel (Microsoft Corporation, Redmond, WA) for analysis. Each void event recorded by the sensor of ≥0.2 g within the 24-h time frame was calculated for each animal. Total drink volume was recorded through the CLAMS volumetric drink monitor. One-way repeated-measures analysis of variance (ANOVA) analyses were used across all SCI animal groups using SigmaStat software (v3.5; Systat Software Inc., San Jose, CA), where significance was determined for p < 0.05.

Data files for AVP, ANP, and creatinine were exported from SoftMax Pro to Microsoft Excel (Microsoft Corporation) for analysis. Samples and standards were averaged to create a standard curve to determine protein concentration. Because of combined variability and sample size, terminal protein concentrations were compared to their normalized baseline level for statistical analysis. Signed-ranks tests were used to compare normalized baseline to terminal protein concentrations, where *p* < 0.05 was considered as having statistical significance. A two-way ANOVA was used for western blot analysis to determine statistically significant interactions between antibodies used and terminal time points. Values were considered significant if *p* < 0.05.

## Results

Nine animal groups in total consisted of six post-SCI recovery time points (3, 7, 14, 28, 42, and 84 dpi) and three post-sham laminectomy time points (3, 14, and 42 dpi). Before contusion injuries, pre-injury baseline data were collected, including: 24-h urine output and collection/drink volume, blood draws, and BBB assessments. All animals scored ≥19 on the BBB scale, indicating that there were no past or pre-existing motor deficits. *Post hoc* analyses were carried out between the six SCI groups and revealed no significant differences for injury parameters (force, displacement) or 4-dpi residual urine volume emptied by Credé (Table 1). However, there were significant differences among BBB scores, where both the 3- and 7-dpi animal groups received significantly lower scores at terminal time points compared to 14-, 28-, 42-, and 84-dpi animals (*p* < 0.05), a finding consistent with the timing of previous locomotor outcome data showing some limited recovery during the first 2 weeks post-contusion.^6^

### Twenty-four-hour urine volume

**FIG. 1.**
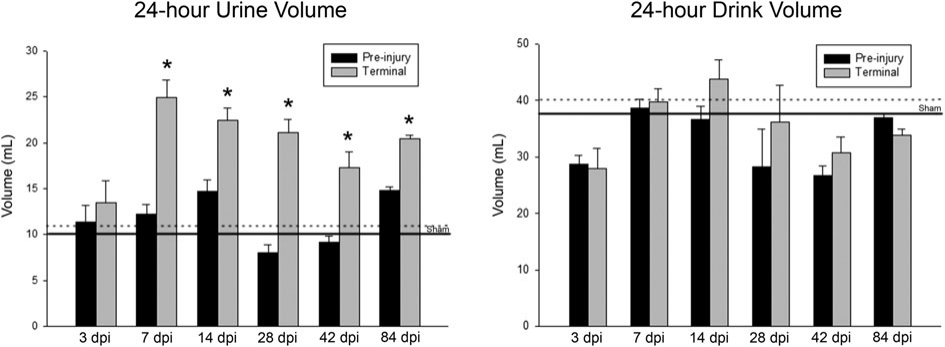
Metabolic cage data summary. Total 24-h urine volume output (left plot) indicates a statistically significantly increase at the 7-, 14-, 28-, 42-, and 84-dpi time points, but not at 3 dpi, relative to their pre-injury baseline and shams. Total 24-h drink volume (right plot) indicates the absence of any statistically significant differences over time relative to pre-injury levels as well as shams. Values are represented as means with SEM bars included (*p < 0.05). Data collected from three sham animal groups were combined because no significant differences were found (solid horizontal line represents pre-injury time point; dotted line post-injury terminal time point). dpi, days post-injury; SEM, standard error of the mean.

Both urine production and drink volume were measured weekly across 24 h to determine presence/absence of SCI-induced polyuria. Before contusion injuries, each animal's baseline was recorded as a further control (in addition to sham groups) for each time-point group. SCI-induced polyuria was present in all SCI animal groups except for 3 dpi (Fig. 1). By 7 dpi, each animal group had a statistically significant increase in 24-h urine volume (*p* < 0.05). Neither of the time-point groups after 3 dpi (7, 14, 28, 42, and 84 dpi) were significantly different from each other in urine output. Despite the significant increase in urine production among all SCI animal groups apart from 3 dpi, there were no significant increases in drink volume across any of the groups, a finding consistent with previous studies.^6,9,10^ Note that no signs of dehydration (skin turgidity, reduced urine volume, urine color change reflecting higher concentration, or porphyrin staining of the eyes and nose) were observed during the daily nursing care of rats post-SCI.

### Enzyme-linked immunosorbent assay for urinary atrial natriuretic peptide and serum arginine vasopressin

**FIG. 2.**
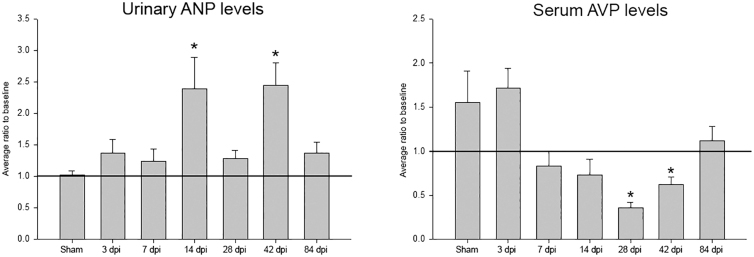
Urinary ANP and serum AVP. Both urinary ANP and serum AVP levels were normalized to their pre-injury baseline concentrations. At 14 and 42 dpi, urinary ANP/creatinine levels were significantly increased compared to pre-injury baseline levels. However, sham and 3-, 7-, or 84-dpi groups did not demonstrate any statistically significant changes in urinary ANP/creatinine levels. Serum AVP concentrations were significantly decreased at 28 and 42 dpi, but not 84 dpi. Error bars represent SEM. *Significantly different from pre-injury baseline, *p* < 0.05. ANP, atrial natriuretic peptide; AVP, arginine vasopressin; dpi, days post-injury; SEM, standard error of the mean.

The importance of ANP in water balance, specifically in relation to cardiovascular changes, is well established.^32–34^ Thus, ELISA was used to determine when and whether alterations in urinary ANP occurred after SCI, as previously reported,^9,10^ using urine samples from the 24-h metabolic cage assessments. Urinary ANP levels were found to be significantly increased at both 14 and 42 dpi relative to baseline levels (Fig. 2). Because blood pressure and water balance are heavily affected by circulating AVP, ELISA was also performed to obtain serum concentrations of AVP at pre-injury and terminal time points. Serum AVP levels were significantly lower at two of the chronic time points relative to pre-injury baseline concentrations (Fig. 2).

### Western blot of kidney tissues

**FIG. 3.**
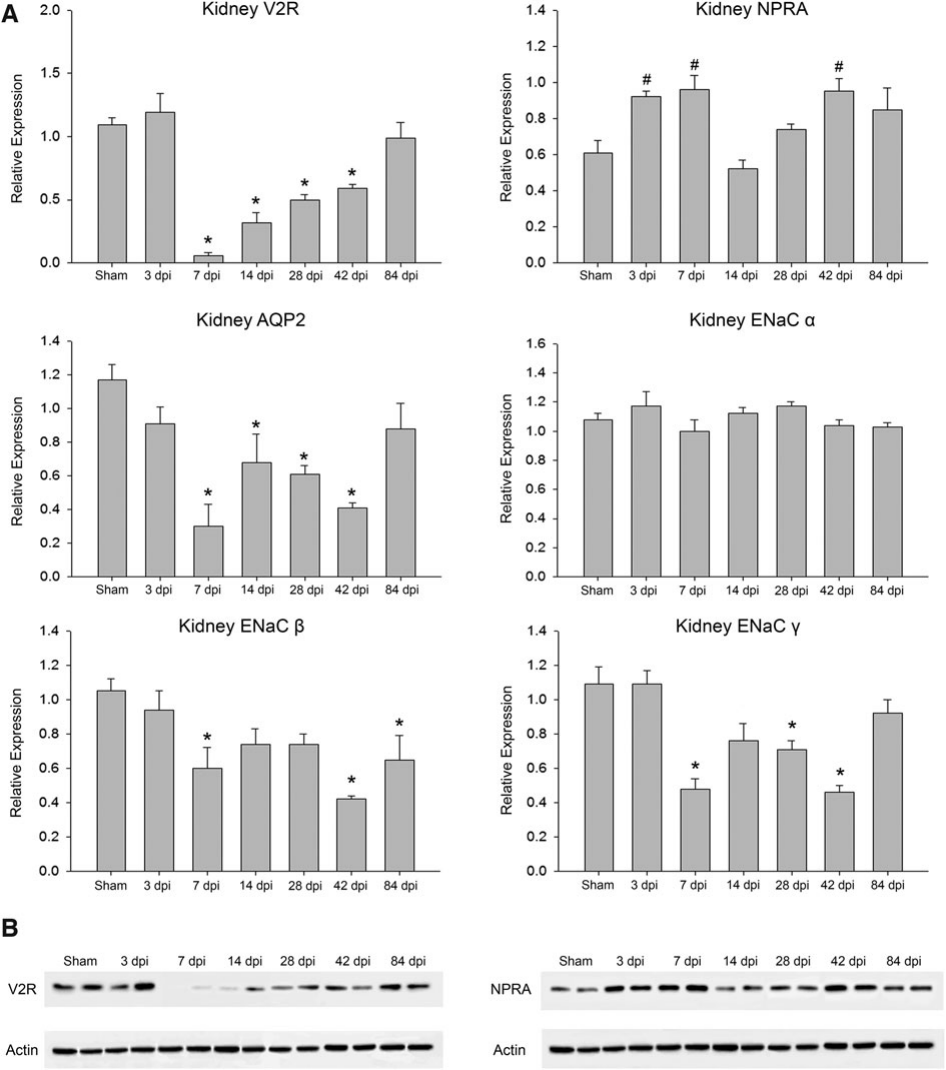
Kidney tissue western blot summary of results. As shown graphically in (**A**), relative expression of vasopressin 2 receptor (V2R), aquaporin 2 (AQP2) channel densities, and epithelial sodium channels (ENaC β- and γ-subunits) from kidney tissue were significantly depressed at various time points compared to surgical sham animals, as well as 3 dpi (*). In contrast, relative expression of natriuretic peptide receptor A (NPRA) in kidney was significantly lower at 14 dpi compared to 3, 7, and 42 dpi (#). Representative examples of western blots showing expression levels are provided in (**B**) for V2R and NPRA. dpi, days post-injury.

Western blot analysis was performed on kidney tissue from 6 randomly selected animals per group to visualize relative expression density of NPRA, V2R, AQP2, and ENaC (α-, β-, and γ-subunits; Fig. 3). For analysis of the time-point factor overall (regardless of receptor or channel), both sham and 3 dpi were significantly different from all other time points, except for the 84-dpi group, which differed from 3 dpi, but not shams. Relative expression of kidney V2R was significantly decreased by 7 dpi and remained depressed at each subsequent time point except for 84 dpi. The same pattern occurred for kidney AQP2 expression, which is directly related to V2R expression. For both V2R and AQP2, 7 dpi was significantly lower than several of the later time points. In contrast, density of NPRA expression was significantly higher at 3, 7, and 42 dpi relative to only 14 dpi. In addition, because the α-, β-, and γ-subunits form together to make ENaC, western blot analysis was done on all three.

Although the density of ENaC α expression was unchanged at all time points compared to time-point–matched sham groups, both ENaC β and γ expression densities were decreased after SCI at several time points, beginning at 7 dpi. All pair-wise multiple comparison procedures (Bonferroni's *t*-test) revealed a significant effect for only the ENaC α-subunit relative to the other five antibodies (*p* < 0.001), reflecting the lack of a time-point effect for relative expression of ENaC α.

### Arginine vasopressin labeling in hypothalamus

**FIG. 4.**
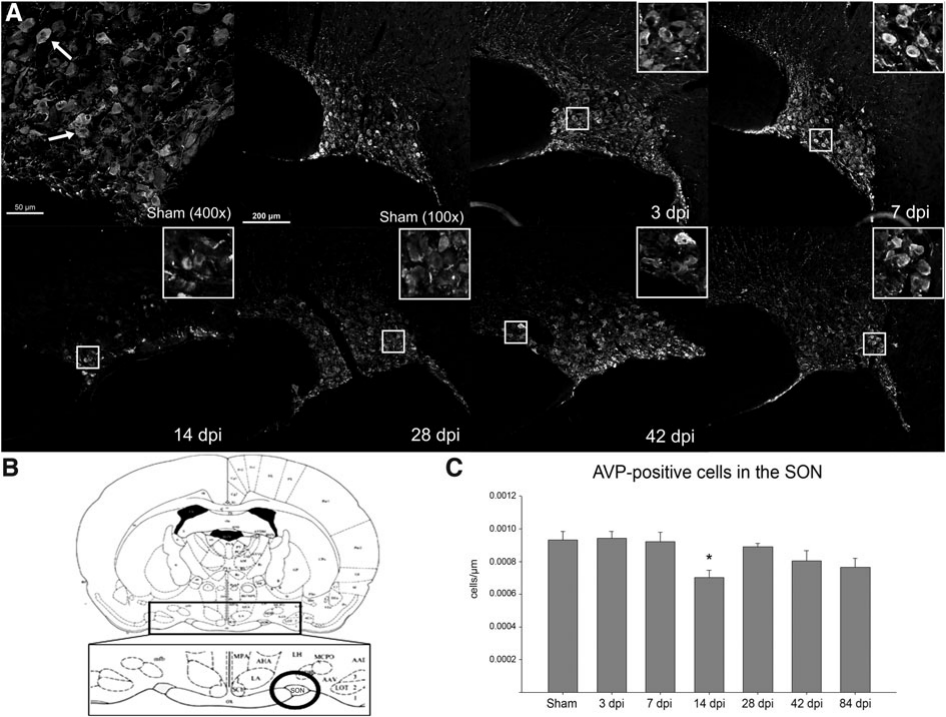
The supraoptic nucleus (SON). A representative section showing AVP-labeled cells in the SON at each time-point group is provided in (**A**), including a higher magnification of the sham group example for visualization of labeled (arrow) cells. In (**B**), a modified plate from the Rat Brain Atlas illustrates the location of the SON within the hypothalamus.^29^ Magnified insets are included to show examples of AVP labeling. The average number of AVP-labeled cells per area in the SON for each time-point group is presented graphically in (**C**). The only time point after SCI that demonstrated a statistically significant different number of AVP-positive cells in the SON compared to the sham group was at 14 dpi (*p < 0.05, error bars represent SEM). SON: sham (*n* = 9), 3 dpi (*n* = 7), 7 dpi (*n* = 7), 14 dpi (*n* = 8), 28 dpi (*n* = 6), 42 dpi (*n* = 7), and 84 dpi (*n* = 6). AVP, arginine vasopressin; dpi, days post-injury; SCI, spinal cord injury; SEM, standard error of the mean.

**FIG. 5.**
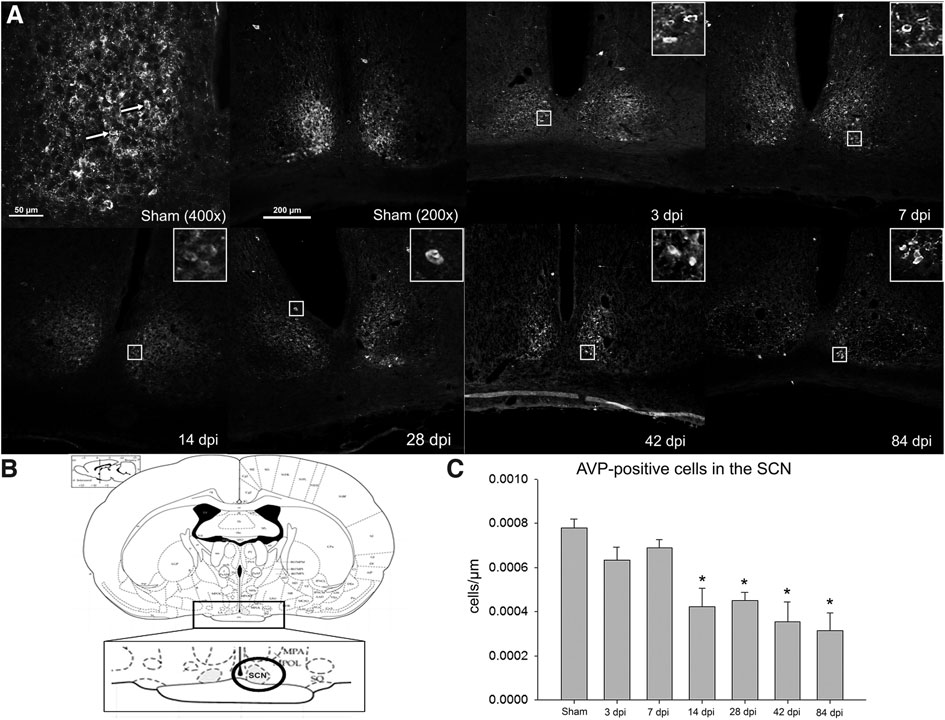
The suprachiasmatic nucleus (SCN). A representative section showing AVP-labeled cells in the SCN at each time-point group is provided in (**A**), including a higher magnification of the sham group example for visualization of labeled (arrow) cells. Magnified insets are included to show examples of AVP labeling. In (**B**), a modified plate from the Rat Brain Atlas illustrates the location of the SCN within the hypothalamus.^29^ The average number of AVP^+^ cells/μm in the SCN (shown in **C**) are significantly decreased at the 14-, 28-, 42-, and 84-dpi time points, but not at 3 or 7 dpi (*p < 0.05, error bars represent SEM). SCN: sham (*n* = 3), 3 dpi (*n* = 4), 7 dpi (*n* = 3), 14 dpi (*n* = 4), 28 dpi (*n* = 3), 42 dpi (*n* = 3), and 84 dpi (*n* = 5). AVP, arginine vasopressin; dpi, days post-injury; SEM, standard error of the mean.

The number of AVP-positive cells in the SON was examined in all groups, and the only difference found was for the 14-dpi time point, which was significantly fewer relative to shams (Fig. 4). Although the SON was the main target of this study, both PVN and SCN neurons that were present within the same tissue slices were also examined. However, because of their relative size and location relative to the SON, the overall cellular sample size was smaller. Within the PVN, there were no significant decreases in observed AVP-positive cells at any time points compared to sham animals (data not shown). However, the average AVP-positive cells per area within the SCN was significantly decreased at 14 dpi and remained that way at all later time points (Fig. 5).

### White matter sparing

**FIG. 6.**
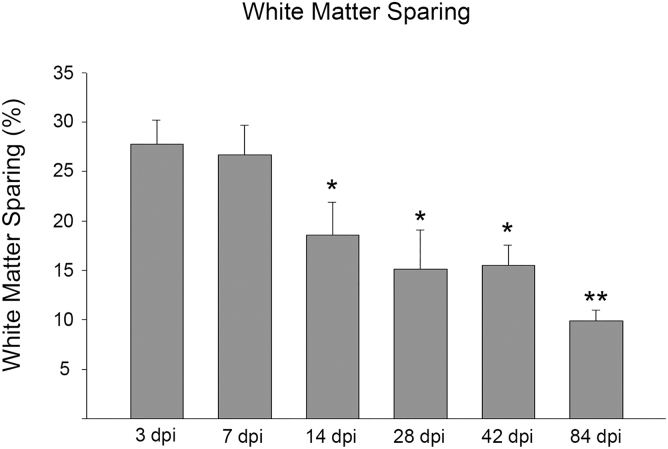
White matter sparing (WMS). A significant decrease in WMS occurred from the 14-dpi time point onward. At 84 dpi, there was a further significant decrease in WMS relative to 42 dpi (*vs. 3 and 7 dpi, *p* < 0.05; **vs. 42 dpi, *p* < 0.05). Error bars represent SEM. AVP, arginine vasopressin; dpi, days post-injury; SCN, suprachiasmatic nucleus; SEM, standard error of the mean.

WMS, an indicator of injury severity, was quantified for all SCI animal groups. As shown in Figure 6, the WMSs for both 3 and 7 dpi were not different from each other. There was a statistically significant decrease in WMS at the 14-dpi time point and beyond compared to both 3 and 7 dpi. Also, WMS was further decreased by the 84-dpi time point, indicating continued progression of damage at the lesion epicenter beyond 6 weeks post-contusion.

## Discussion

SCI-induced polyuria has been established as a major systemic issue that arises in both human and pre-clinical animal SCI models.^6,11,35^ Previous data from our lab have shown that several key hormones and receptors, including AVP, ANP, V2R, and NPRA, are associated with mechanisms underlying SCI-induced polyuria.^9,10^ The current data reveal the timing of these biomarker changes within the kidney itself with some additional contributions by AVP-positive neurons in the hypothalamus. Together, the current results illustrate both peripheral and supraspinal effects within one of many internal organ systems post-SCI and demonstrate the impact of disrupting the body's homeostatic mechanisms on functional outcome and, ultimately, quality of daily living.

Animals in the current study presented with polyuria by 7 dpi and lasted through the longest time point examined (84 dpi). This result differs from the emergence of deficits in locomotor function, which appear immediately because of initial spinal shock, but then partially recovers and plateaus after several weeks at a level that is known to correlate with extent of injury^36,37^ (and as shown by the BBB scores in Table 1). The fact that polyuria onset is slightly delayed and maintains at a steady state despite continued progression of damage at the lesion epicenter (WMS data) indicates that the underlying mechanisms are likely secondary to damage of the spinal cord itself. Noteworthy is that this increase in urine volume was not matched with an increase in drink volume, indicating that the source of excess fluids was not attributable to greater intake. In addition, typical signs of dehydration were not present given that all rats were visually inspected and tended to daily. It remains unclear as to the source of the additional urine being produced and voided across all time points. Previous experiments investigating fecal water weight resulted in a difference in acute, but not chronic, time points, suggesting that water does not come from any changes in fluid content within the feces.^6^

Serum osmolality was also previously tested and found to be significantly increased after SCI.^9^ Increased osmolality was in the direction that should have stimulated the release of more AVP to reabsorb more water, which did not occur because of an overall disruption of homeostatic mechanisms. Thus, animals may have been slightly underhydrated, but not enough to stimulate thirst or show any of the typical signs related to dehydration. In fact, restriction of fluids at night is done by many SCI persons to limit the number of awakenings for nighttime catheterizations. Desmopressin, an available option to treat nocturnal polyuria, has limited success for persons with SCIs,^14,15^ indicating that further studies are needed.

Urinary ANP and serum AVP levels for the various SCI animal time-point groups were significantly altered in the direction that would have produced polyuria at various 14- to 42-dpi time points, but did not match the onset (7 dpi) and duration of the overproduction of urine that was measured. These findings signify that changes in serum levels of ANP and AVP are variable and not static, which implicates other important factors, such as corticosterone (increased urinary levels previously shown at 14 dpi) and/or key receptors/channels (V2R, NPRA, AQP2, and ENaC) in the kidney (altered levels previously shown at the 70-dpi time point).^9,10^ Although AVP and ANP are key regulators of water and solute balance and likely play a major role in SCI-induced polyuria, they are not the only factors involved in urine production. The result that the significant alterations did not match up with chronic polyuria is indicative that other mechanisms and biomarkers are involved.

In the current study, western blot analysis of fresh kidney tissue at each time point after SCI revealed significant changes at many post-SCI, but not sham laminectomy or 3-dpi, time points for one or more of the receptors/channels examined. Both V2R and AQP2, which work together, were significantly decreased starting at 7 dpi, but reverted to sham levels by 84 dpi. Given that polyuria is still present at this time point, it is likely that these levels reflect constant variability within the homeostatic water balance system rather than recovery to baseline levels. Later time points beyond 84 dpi would be necessary to address this possibility.

For NPRA, significant changes were present in several time points relative to 14-dpi, but not sham and 3-dpi, groups. The timing of the fluctuations, when considered with the ANP ELISA data, indicates that different compensatory mechanisms may be at play. The fluctuations of NPRA levels and ANP post-SCI could reflect its functional role on two different systems. NPRA levels could be a response to low blood volume that is often noted after SCI and/or attributable to disruption of urinary regulation.^38–41^ Increased NPRA has been shown to be associated with downregulation of ANP.^12^ SCI often results in hypotension, which may result in an increase in NPRA, causing a significant decrease in blood pressure.^40^ Low levels of ANP are associated with hypertension, and the inverse is true that an increase in ANP leads to hypotension.^42^ Further research will be needed to better understand these multi-system effects. Note that, previously, these receptors/channels were shown to be positively impacted with activity-based recovery training.^10^ However, training benefits multiple locomotor and non-locomotor systems, including functions of both the urinary tract and cardiovascular system. ^4,10,30,43,44^

There was no variation in the relative density of the ENaC α-subunit after SCI, whereas both ENaC β and γ were significantly decreased at several time points. Differences in kidney expression levels of the various ENaC subunits post-SCI remain unclear. ENaC α is critical for ENaC pore formation, given that it can form a functional channel on its own, whereas ENaC β and γ are responsible for membrane surface expression (functionality).^45^ Channels formed only with the α-subunit form less functional channels than with all three subunits together. Further, the β- and γ-subunits are more molecularly similar, making it clear that there is a distinct difference between the β-/γ- versus α-subunit.^46^ Note that studies investigating the distinction between the β- and γ-subunit concluded that the γ-subunit is more important than the β-subunit for cell membrane surface trafficking and expression, thus overall channel functionality.^47,48^

Previously, we found that both AVP and ANP, in addition to kidney V2R, NPRA, AQP2, and ENaC levels, were altered 2 weeks post-SCI and were significantly altered at 10 weeks. Interestingly, findings throughout this study include these same fluctuations in both AVP/ANP as well as several of the receptors, but several of the time points in between revert to sham levels. These results suggest that the system may be constantly trying to correct itself (as noted by “normal” levels of AVP/ANP and receptor densities).

However, polyuria is present at every time point starting at 7 dpi, which further suggests that water-balance homeostasis is never fully achieved after SCI and there are likely multiple systems involved, including others not examined here such as the renin angiotensin aldosterone system (RAAS) and/or corticosterone levels.^49^ The RAAS is essential for fluid balance and cardiovascular health (i.e., blood pressure regulation), which are commonly impacted by SCI.^50,51^ The extent of how RASS is affected by SCI is currently unknown and therefore should be examined further in future studies.^52^ Note that the ENaC is regulated by aldosterone,^53,54^ a critical part of RAAS,^49^ and both the ENaC β- and γ-subunits in the kidney were significantly decreased after SCI.

To further identify possible mechanisms contributing to SCI-induced AVP dysfunction, the density of AVP-positive cells in SON, SCN, and PVN of the hypothalamus was examined. The critical role of AVP in controlling water/salt balance is well established.^55,56^ AVP is also known to escalate inflammation, which further contributes to neuronal disruptions after traumatic brain injury,^57^ in addition to its association with SCI-induced polyuria.^9,10^ Whereas SON analysis revealed a significant decrease in AVP-labeled cells at 14 dpi, there were no other time points that demonstrated significant differences compared to surgical sham animals. Previously, a decrease in serum AVP was found as early as 2 weeks post-contusion, whereas this study found a significant decrease in AVP at 28 and 42 dpi, with a recovery at 84 dpi. However, western blot analysis revealed that kidney V2R levels were decreased as early as 7 dpi. This finding suggests that there is a significant change in V2R regardless of serum AVP levels and further reveals the instability of this system after SCI.

Under normal physiological conditions, the sympathetic nervous system works together with neuroendocrine and hormonal control in both central and peripheral homeostatic mechanisms. After SCI, this balance is disrupted and causes a cascade of dysfunction that involves hormonal and neuroendocrine changes, which subsequently lead to physical symptoms such as orthostatic hypotension and SCI-induced polyuria.

In contrast to the SON, the number of AVP-labeled cells in the SCN was significantly lower in all SCI animal groups starting at 14 dpi. The SCN is closely tied with circadian control, which is very important for AVP release.^58^ Recent studies have shown significant disruptions of circadian rhythmicity post-SCI, including activity, body temperature, clock gene expression, and corticosterone production.^59,60^ Clock genes, which are, in part, responsible for many biological functions, such as sleep/wake cycles, the autonomic nervous system, body temperature, and gastrointestinal motility,^61–63^ are also functions regulated by the SCN and are all disrupted post-SCI.^64–66^ The contribution of the SCN to SCI-induced polyuria likely relates to the diurnal variation of AVP production, given that clinical studies have identified the loss of this pattern as being responsible for the emergence of nocturia after SCI.^5,11,67^

For the PVN, no significant differences in AVP-labeled cells were found post-SCI. Note that the entire PVN was not examined, and, based upon its location relative to the SON, fewer areas were analyzed, which reduces the possibility of finding any differences that may exist. The PVN is only one of three regions where AVP is produced,^18^ and to a lesser extent than the SON,^68^ but a full analysis of the entire region should be done in future experiments to determine definitively whether SCI impacts this area of the hypothalamus. Given that the data taken from the PVN analysis are limited, it is pilot in nature and further studies are needed to make any conclusions. However, an increase in animals and PVN cell counts will likely not yield significant results given that the SON yielded a significant decrease in AVP-positive cells at only one time point.

Taken together, the results from this study provide further evidence that SCI is a complex and progressive injury, which includes changes in various metabolic peptides (AVP, ANP, V2R, NPRA, and ENaC) that contribute to the development and maintenance of SCI-induced polyuria. One study limitation is that the RAAS was not examined. As mentioned above, the RAAS plays a major role in homeostatic fluid balance and should be a target for future studies not only for SCI-induced polyuria, but also for cardiovascular health. Future studies that target individual elements alone or in combination, including the RAAS, may elucidate each of their contributions toward SCI-induced polyuria and help identify potential therapeutic targets beyond desmopressin, which has limited efficacy (such as hyponatremia^15^ and restricted use on patients ≥65 years of age^69^) and is likely, as the current data suggest, not the only contributing factor related to SCI-induced polyuria.

## Abbreviations Used

ANOVA: analysis of variance
ANP: atrial natriuretic peptide
AQP2: aquaporin-2
AVP: arginine vasopressin
BBB: Basso-Beattie-Bresnahan
CLAMS: Comprehensive Lab Animal Monitoring System
dpi: days post-injury
ELISA: enzyme-linked immunosorbent assay
ENaC: epithelial sodium channels
IHC: immunohistochemistry
NIH: National Institutes of Health
NPRA: natriuretic peptide receptor-A
PFA: paraformaldehyde
PVN: paraventricular nucleus
RAAS: renin angiotensin aldosterone system
SCI: spinal cord injury
SCN: suprachiasmatic nucleus
SON: supraoptic nucleus
TBST: Tris-buffered saline with Tween 20
WMS: white matter sparing
V2R: vasopressin-2 receptor
